# Association of Soluble Tumor Necrosis Factor-Related Apoptosis-Inducing Ligand (TRAIL) with Central Adiposity and Low-Density Lipoprotein Cholesterol

**DOI:** 10.1371/journal.pone.0058225

**Published:** 2013-03-05

**Authors:** Gloria Brombo, Stefano Volpato, Paola Secchiero, Angelina Passaro, Cristina Bosi, Giovanni Zuliani, Giorgio Zauli

**Affiliations:** 1 Department of Clinical and Experimental Medicine, Section of Internal Medicine, Gerontology and Clinical Nutrition, University of Ferrara, Ferrara, Italy; 2 Department of Morphology and Embryology, University of Ferrara, Ferrara, Italy; 3 Institute for Maternal and Child Health, IRCCS Burlo Garofolo, Trieste, Italy; University of Milan, Italy

## Abstract

**Objective:**

Tumor necrosis factor-Related Apoptosis-Inducing Ligand (TRAIL), in addition to having a prognostic value in patients with cardiovascular disease, seems to interact with adiposity, insulin resistance and other cardiovascular risk factors. However, the results of previous clinical studies, focused on the association of TRAIL with selected metabolic or anthropometric indices were inconclusive. The aim of this study was to further investigate how soluble TRAIL concentrations independently correlate with major cardiovascular risk factors, including lipid, glycemic and anthropometric features.

**Materials/Methods:**

We examined the associations between serum soluble TRAIL concentrations, measured by ELISA, and lipid, glycemic and anthropometric features in 199 subjects recruited at our Metabolic Outpatient Clinic.

**Results:**

Soluble TRAIL concentrations had a significant and direct correlation with total cholesterol (*p* = 0.046), LDL-cholesterol (*p* = 0.032), triglycerides (*p* = 0.01), body mass index (*p* = 0.046), waist circumference (*p* = 0.008), fat mass (*p* = 0.056) and insulin (*p* = 0.046) and an inverse correlation with HDL-cholesterol (*p* = 0.02). In multivariable regression analyses adjusted for potential confounders (age, gender, C-reactive protein, HDL-cholesterol, triglycerides, waist circumference, and insulin), TRAIL levels continued to have an independent correlation with LDL-cholesterol and waist circumference (r^2^ = 0.04).

**Conclusions:**

Serum TRAIL levels were weakly but significantly and independently associated with waist circumference, a marker of visceral adiposity, and with LDL-cholesterol. Further studies are needed to clarify the biological basis of these relationships.

## Introduction

Tumor necrosis factor (TNF)-Related Apoptosis-Inducing Ligand (TRAIL) is a member of the TNF superfamily, which is either expressed as a transmembrane protein on the cell surface of a variety of cell types or is released as a soluble protein [1]. TRAIL is capable of inducing apoptosis in cancer cells and seems to be involved in tumor suppression and immune cell homeostasis [2]. However, TRAIL also seems to be active on vascular cells where it exerts anti-inflammatory and anti-atherosclerotic activity *in vitro* [3], [4] and in animal models [5], [6]. In agreement with these preclinical observations on the relationship between TRAIL and cardiovascular disease, some clinical studies found that in patients with coronary artery disease [7]–[9] TRAIL levels tend to be lower and inversely correlated with biomarkers of myocardial damage and with C-reactive protein [8], a strong predictor of future cardiovascular events [10]. In addition, in patients with acute myocardial infarction [8] or advanced heart failure [11], [12], lower levels of soluble TRAIL predicted the risk of death or congestive heart failure over the follow-up. Finally, we have recently demonstrated that in older patients with chronic cardiovascular disease low levels of TRAIL were associated with increased risk of all-cause and cardiovascular mortality over a period of six years [13].

Globally taken, these studies suggested that in patients with cardiovascular disease lower concentrations of circulating TRAIL can be considered as a negative and independent prognostic factor. Nevertheless, clinical correlates and biological factors involved in the modulation of the variability of serum TRAIL concentrations, particularly in subjects free of cardiovascular disease or acute inflammatory conditions, have not been elucidated so far. Some studies suggested an interaction of TRAIL levels with adiposity, insulin resistance and metabolic indices. In apparently healthy individuals TRAIL concentrations were associated with body composition and serum lipid levels, especially total body fat in men and LDL-cholesterol in women [14]. In another sample of subjects free of cardiovascular and metabolic disease TRAIL was positively related to fat mass and waist circumference [15], whereas in patients with type 2 diabetes a significant correlation between TRAIL levels and body mass index, insulin resistance and triglycerides levels has been reported [16].

On these bases, the purpose of this study was to further investigate how soluble TRAIL concentrations correlate with body composition features and metabolic cardiovascular risk factors, especially with lipid and glycemic parameters and anthropometric indices.

## Methods

### Study Population

A total of 199 subjects were consecutively recruited from the Metabolic Outpatient Clinic of the Section of Internal Medicine, Gerontology and Clinical Nutrition (S. Anna University-Hospital, Ferrara, Italy). Patients attended the Metabolic Outpatient Clinic for any obesity-related metabolic disorder, including overweight, dyslipidemia and metabolic syndrome. In order to minimize the effect of life-style modification and/or new medication effect, only patients attending the clinic for the first time were included in the study. No other exclusion criteria were applied for patients selection.

### Biochemical Measures

Venous blood samples were obtained from subjects after a 12-hour fast. Aliquots of serum were stored at −80°C and were not thawed until analyzed. Serum TRAIL was measured in duplicate by specific, commercially available ELISA kit (R&D Systems, Minneapolis, MN), in accordance with the manufacturer’s instructions and analyzed with an ELISA reader at 450 nm. Sensitivity of the assay was 2.86 pg/ml and the intra- and inter-assay coefficients of variation (CV) were 3.9% and 6% respectively and the upper limit of detection was 1000 pg/ml. Total cholesterol and high-density lipoprotein (HDL) cholesterol were determined using enzymatic colorimetric method (Roche Diagnostics, Mannheim, Germany), sensitivity was 3 mg/dl. Low-density lipoprotein (LDL) cholesterol was estimated using Friedewald equation. Triglycerides were determined using enzymatic colorimetric method (Roche Diagnostics, Mannheim, Germany), sensitivity was 4 mg/dl. Glycemia was measured using enzymatic colorimetric method based on Trinder reaction (Far, Verona, Italy). Sensitivity was 3 mg/dl and the intra- and inter-assay CV were 2.5 and 2.7 respectively. Insulin was determined using an enzyme-linked immunosorbent assay (Mercodia, Uppsala, Sweden). Sensitivity was 0.07 mU/l and intra- and inter-assay were 4.9 and 2.8 respectively. Homeostasis Model Assessment (HOMA) index was used as an indicator of insulin resistance [17]. High-sensitivity C-reactive protein (hs-CRP) was measured using an enzymatic colorimetric method (Roche Diagnostics, Mannheim, Germany). Sensitivity of the method was 0.03 mg/l and the intra- and inter-assay CV were 0.8 and 4.1 respectively.

### Anthropometric Indices

Body mass index (BMI, Kg/m^2^) was calculated from a subject’s weight and height, measured using objective standard techniques. Subjects were weighed barefoot and with minimal clothing. Waist circumference was measured at midway between the lowest rib and the iliac crest. Body composition (Fat Mass, Fat Free Mass, Body Cell Mass) was determined by bioelectrical impedance analysis (BIA) using a BIA instrument (Human-Im Plus II). This method is based on the determination of the resistance that a human body opposes to electricity. The BIA measurements were carried out at least after a 4-hour fast and consisted in a current at five different frequencies (5, 10, 50, 100 and 250 kHz) that passed between surface electrodes placed on hand and foot.

### Other Measures

Socio-demographic characteristics of the study population included age and gender. History of smoking (categorized as current, former and never smoker) and medication use was ascertained from the baseline interview. The prevalence of specific medical conditions was established using standardized criteria that combined information from baseline interview, medical records, physical examination and blood test results. Disease categories were: hypertension, type 2 diabetes, coronary heart disease (angina and acute myocardial infarction), cerebrovascular disease (stroke and/or transient ischemic attack), peripheral arterial disease and metabolic syndrome (diagnosed by the NCEP-ATPIII criteria).

### Statistical Analysis

For descriptive purpose, selected clinical and metabolic characteristics of the study population were compared according to tertiles of plasma TRAIL levels (40.3–72.4 pg/ml, n = 66; >72.4–93.1 pg/ml, n = 66; >93.1–178.9 pg/ml, n = 66), using a χ^2^ test and ANOVA model for categorical and continuous variables, respectively. Continuous variable with skewed distribution were log-transformed in order to approssimate a normal distribution. We used Pearson’s correlation analysis to estimate the association between TRAIL and anthropometric and metabolic characteristics. In order to select the independent correlates of soluble TRAIL levels, multivariable analysis has been re-performed using multiple linear regression analysis predicting serum TRAIL levels according to different metabolic and anthropometric characteristics. All models were initially adjusted for age, gender, and CRP. A final fully adjusted model was also performed including all variables statistically associated with TRAIL at univariate analysis. When the correlation coefficient between 2 variables was greater than 0.5, only one of the two variables was included in the final multivariable model, namely the variable with the higher correlation coefficient with TRAIL. Finally, in order to avoid overparametrization and collinearity, unnecessary variables (independent variables not statistically associated with the dependent variable) were removed from the final model using a stepwise backward selection technique (p for removal 0.1). All analyses were performed using Stata 11.0 for Windows (College Station, TX: Stata Corporation).

## Results

**Table 1** presents some demographic and metabolic characteristics of the study population. High total cholesterol levels were defined for values ≥200 mg/dl, high LDL-cholesterol for values ≥130 mg/dl, low HDL-cholesterol for values ≤50 mg/dl in women and ≤40 mg/dl in men, high triglycerides for values ≥150 mg/dl. Almost all the patients (98.5% of the study population) carried out at least one metabolic risk factor and 90.5% had 2 or more conditions.

**Table 1:** Metabolic characteristics of study participants.

Characteristics	
Age (mean ± SD)	57.3±11.5
Female (N, %)	95 (47.7)
Male (N, %)	104 (52.3)
Waist circumference, cm (mean ± SD)	101.4±14
Overweight (N, %)	82 (41.2)
Obesity (N, %)	91 (45.7)
Impaired fasting glycemia (N, %)	49 (24.6)
Type 2 diabetes (N, %)	23 (11.6)
High total cholesterol (N, %)	108 (54.3)
Low HDL-cholesterol (N, %)	80 (40.2)
High LDL-cholesterol (N, %)	99 (49.8)
High triglycerides (N, %)	62 (31.2)
≥1 metabolic risk factor (N, %)	196 (98.5)
≥2 metabolic risk factors (N, %)	180 (90.5)

**Table 2** and **Table 3** present the socio-demographic characteristics, anthropometric indices, health conditions, biochemical parameters and medications of the study population according to tertiles of soluble TRAIL concentrations. Subjects with higher TRAIL levels tended to be older and had significantly greater BMI (*p* = 0.009), waist circumference (*p* = 0.003) and fat mass (*p* = 0.014). In addition they had higher triglycerides (*p* = 0.038), insulin (*p* = 0.003) and HOMA index (*p* = 0.016) and lower HDL-cholesterol (*p* = 0.009). There was no significant association between TRAIL and other participants’ characteristics including gender, blood pressure, fat-free mass, body cell mass, total cholesterol, LDL-cholesterol, CRP, health conditions and selected medications use.

**Table 2:** Characteristics of study participants according to TRAIL distribution.

Characteristics	Tertiles of plasma TRAIL levels (pg/ml)			*P*
	40.3–72.4	>72.4–93.1	>93.1–178.9	
	(n = 66)	(n = 66)	(n = 66)	
Sex, N (%)				
Female	34 (51.5)	28 (42.4)	32 (48.5)	
Male	32 (48.5)	38 (57.6)	34 (51.5)	0.567
Age, mean ± SD	54.9±12.6	59.5±10.9	57.6±10.7	0.071
Smoking, N (%)				
Former	29 (43.9)	33 (50.0)	21 (31.8)	
Current	5 (7.6)	5 (7.6)	13 (19.7)	0.072
Health conditions, N (%)				
Hypertension	21 (31.8)	29 (43.9)	27 (40.9)	0.331
Type 2 diabetes	6 (9.1)	10 (15.2 )	7 (10.6)	0.528
Coronary heart disease	2 (3.1)	4 (6.1)	5 (7.6)	0.522
Cerebrovascular disease	4 (6.2)	2 (3.0)	4 (6.1)	0.650
Peripheral arterial disease	5 (7.7)	4 (6.1)	10 (15.2)	0.169
Metabolic syndrome	14 (21.2)	18 (27.3)	24 (36.4)	0.151

**Table 3:** Metabolic characteristics of study participants according to TRAIL distribution.

Characteristics	Tertiles of plasma TRAIL levels (pg/ml)			*P*
	40.3–72.4	>72.4–93.1	>93.1–178.9	
	(n = 66)	(n = 66)	(n = 66)	
Anthropometric indices, mean ± SD				
Body mass index, Kg/m^2^	29.9±6.7	30.1±5.7	33.0±7.2	0.009
Waist circumference, cm	97.6±13.6	101.0±11.8	105.8±15.2	0.003
Fat mass, Kg	29.4±13.4	31.0±12.1	35.9±14.0	0.014
Fat-free mass, Kg	51.8±8.5	51.8±7.7	53.3±11.1	0.556
Body cell mass, Kg	24.8±4.4	24.7±4.5	25.2±4.7	0.786
Biochemical parameters, mean ± SD				
Total Cholesterol, mg/dl	205.9±42.5	207.2±40.1	216.9±49.7	0.300
HDL-Cholesterol, mg/dl	53.9±13.3	48.6±16.2	46.1±14.7	0.009
LDL-Cholesterol, mg/dl	128.2±39.9	132.5±34.2	140.4±45.3	0.209
Triglycerides, mg/dl	121.9±52.1	129.7±60.6	150.4±80.7	0.038
Glycemia, mg/dl	95.5±22.2	100.9±23.1	97.3±16.3	0.323
Insulin, mU/l	9.3±7.6	8.6±4.7	13.5±12.5	0.003
HOMA	2.3±2.0	2.2±1.6	3.4±3.7	0.016
C-reactive protein, mg/dl	0.35±0.6	0.27±0.3	0.53±1.5	0.264
Medications, N (%)				
Lipid-lowering therapy	11 (16.7)	12 (18.2)	11 (16.7)	0.958
Oral hypoglycemic agents	3 (4.5)	6 (9.1)	5 (7.6)	0.634
Antihypertensive drugs	20 (30.3)	23 (34.8)	26 (39.4)	0.508
Antiplatelet drugs	6 (9.1)	10 (15.2)	8 (12.1)	0.586

The linear correlations between TRAIL and characteristics of study participants are presented in **Table 4**. Serum TRAIL concentrations showed a significant positive correlation with total cholesterol (*p* = 0.046), LDL-cholesterol (*p* = 0.032), triglycerides (*p* = 0.01) and insulin (*p* = 0.046) and a significant inverse correlation with HDL-cholesterol (*p* = 0.02).

**Table 4:** Partial linear correlation coefficients of serum TRAIL levels and metabolic characteristics of study participants.

Characteristics	TRAIL	
	Coefficient r	*P*
Age	0.095	0.185
Blood Pressure		
Systolic blood pressure, mmHg	0.026	0.722
Diastolic blood pressure, mmHg	0.090	0.216
Lipid parameters		
Total Cholesterol, mg/dl	0.142	0.046
HDL-Cholesterol, mg/dl	−0.165	0.020
LDL-Cholesterol, mg/dl	0.153	0.032
Triglycerides, mg/dl	0.182	0.011
Glycemic parameters		
Glycemia, mg/dl	0.038	0.592
Insulin, mU/l	0.142	0.046
HOMA	−0.009	0.902
C-reactive protein (ln), mg/dl	0.105	0.142

**Figure 1:**
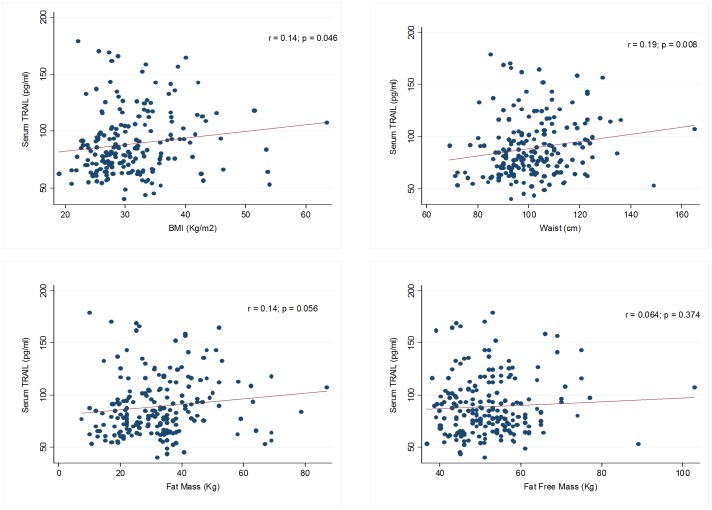
Scatterplots graphs for the correlation between serum TRAIL levels and selected anthropometric indices.

**Figure 1** shows the correlations between anthropometric indices and TRAIL. Serum TRAIL levels had a positive correlation with body mass index (r = 0.14, *p* = 0.046), waist circumference (r = 0.19, *p* = 0.008), and fat mass (r = 0.14, *p* = 0.056), whereas no association was detected with fat-free mass (r = 0.064, *p* = 0.374).

**Table 5** displays correlation matrix of metabolic and anthropometric variables. **Table 6** shows multiple linear regression analysis predicting TRAIL serum level according to different metabolic and anthropometric characteristics. The correlation between TRAIL and HDL-cholesterol, triglycerides and insulin was attenuated and no longer statistically significant after multivariable adjustment whereas the direct association with LDL-cholesterol (β = 0.10, *p* = 0.047) and waist circumference (β = 0.33, *p* = 0.019) persisted even after adjustment for age, gender and other metabolic characteristics.

**Table 5:** Correlation matrix between metabolic and anthropometric characteristics of study participants.

	LDL		Triglycerides		Insulin		Waist		Fat Mass	
HDL	−0.03	0.695	−0.30	<0.001	−0.27	<0.001	−0.30	<0.001	−0.16	0.023
LDL	–	–	0.11	0.139	0.06	0.397	0.05	0.455	0.08	0.262
Triglycerides	–	–	–	–	0.33	<0.001	0.35	<0.001	0.30	<0.001
Insulin	–	–	–	–	–	–	0.49	<0.001	0.43	<0.001
Waist	–	–	–	–	–	–	–	–	0.85	<0.001

**Table 6:** Multivariable linear regression models predicting serum TRAIL levels according to selected metabolic characteristics of study participants.

	Model 1		Model 2		Model 3		Model 4	
	β (SE)	p	β (SE)	P	β (SE)	p	β (SE)	p
HDL-C (mg/dl)	−.32(.14)	0.029	−.32(.14)	0.027	−.17(.15)	0.269	–	–
LDL-C (mg/dl)	.11(.05)	0.035	.11(.05)	0.033	.09(.05)	0.072	.10(.05)	0.047
Triglycerides (mg/dl)	.08(.03)	0.007	.08(.03)	0.008	.05(.03)	0.159	–	–
Waist (cm)	.37(.14)	0.010	.37(.15)	0.011	.20(.17)	0.241	.33(.14)	0.019
Insulin (mU/l)	.48(.22)	0.032	.47(.22)	0.035	.13(.26)	0.616	–	–

Model 1 adjusted for age and gender.

Model 2 adjusted for age, gender and C-reactive protein.

Model 3 adjusted for age, gender, C-reactive protein and all variables included in table.

Model 4 Model 3 with stepwise backward selection of unnecessary variables (p for removal 0.1).

## Discussion

In this study we investigated the relationship of serum soluble TRAIL levels with body composition parameters and metabolic cardiovascular risk factors in a sample of outpatients enrolled at our Metabolic Clinic. We have demonstrated a significant association between TRAIL concentrations and lipid profile, in fact TRAIL had a direct correlation with total cholesterol, LDL-cholesterol and triglycerides and an inverse correlation with HDL-cholesterol. TRAIL was directly and significantly associated with anthropometric indicators of adiposity, including BMI, waist circumference and fat mass. In addition, TRAIL levels were also associated with fasting insulin levels, a strong correlate of adiposity and in particular central obesity. In multivariable analyses, adjusted for potential confounders, we have demonstrated an independent correlation between TRAIL concentrations and LDL-cholesterol and waist circumference, whereas the correlations with HDL-cholesterol, triglycerides and insulin tended to be attenuated and no longer significant. These findings suggest a close and significant association between soluble TRAIL levels and the amount of visceral adipose tissue.

Our study, addressing at the same time the complex interplay of lipid, glycemic and anthropometric features of the study population, expands the contrasting findings of previous clinical studies on the potential association of soluble TRAIL with body composition indices and metabolic parameters. In particular, in a study performed in a sample of apparently healthy adults, serum TRAIL concentrations were correlated in both men and women with BMI, total body fat, lean body mass, total cholesterol and LDL-cholesterol and only in men also with waist-to-hip ratio but the strongest associations were with total body fat in men and LDL-cholesterol in women [14]. Our results suggest that the association of TRAIL with waist circumference and LDL-cholesterol is not mediated or confounded by an inflammatory state or a condition of insulin resistance. Another study, focused on the relationship of TRAIL with obesity and insulin sensitivity in subjects free of cardiovascular and metabolic disease, found a direct association of TRAIL only with fat mass and waist circumference but no significant differences between BMI categories for TRAIL were reported [15]. Instead, in a sample of subjects with type 2 diabetes, serum TRAIL levels were significantly correlated with BMI and also with the HOMA index and triglycerides but no significant associations were found with systolic blood pressure, total cholesterol, LDL-cholesterol and HDL-cholesterol [16].

Our study suggests that TRAIL levels are increased in people with high cardiovascular risk because it’s related to visceral fat and to high-risk lipid profile. These findings seem to be in contradiction with the results of clinical and epidemiological studies, focused on the role of soluble TRAIL in people with cardiovascular disease, that have demonstrated that higher levels of circulating TRAIL can be considered as a protective prognostic factor in term of the risk of future cardiovascular events and mortality [7]–[12].

Globally taken, the results of our and previous studies would suggest that high levels of TRAIL are correlated with traditional cardiovascular risk factors, but in persons with prevalent cardiovascular disease they are associated with fewer cardiac complications and lower mortality. Since both the cellular source of serum TRAIL and the mechanisms of secretion of soluble TRAIL are not fully understood, it is unclear whether the direct correlation of TRAIL concentration with obesity reflects an enhanced synthesis and/or release from adipocytes or the consequence of other biological pathways activated by adipose tissue and/or increased levels of circulating lipoproteins. Nevertheless, the underlying biological explanation for this observed paradox cannot be investigated in our cross-sectional analysis in which the temporal relationship between variables cannot be established. However, some potential mechanisms can be postulated. First, several clinical and epidemiological studies have demonstrated that in the general population obesity and overweight, indexed by body mass index or waist circumference, are related to increased risk of acute cardiovascular disease and heart failure but in patients with prevalent cardiovascular disease they are also strong and independent predictors of improved outcomes and are associated with lower risk of mortality [18], [19]. A potential explanation, in addition to possible confounding effects of characteristics of study population, is that elevated TRAIL levels, regardless of its origin, might protect overweight or obese subjects with cardiovascular disease from further cardiovascular events. A second, and not mutually exclusive hypothesis, is that higher levels of soluble TRAIL might represent an adaptive mechanism finalized to counteract the inflammatory and atherogenetic effect of central adiposity and abnormal lipid profile. Indeed, experimental studies have demonstrated that TRAIL exerts anti-inflammatory and anti-atherosclerotic activity in vitro and in animal models [5]. Nevertheless, additional studies are needed to formally test this hypotheses.

Analyzing the results of this study, some limitations should be considered. The main limitation is the cross-sectional design that doesn’t allow to identify the temporal and causal relations among factors considered. In fact, we found a significant correlation between TRAIL levels and central obesity but we can’t interpret the cause-effect relationship of this association. In addition, the number of subjects recruited was limited and subgroup analyses were not performed because of limited statistical power. Finally, we can’t completely rule out that the results of this study are influenced by confounding factors not considered in our analysis.

In conclusion, we have demonstrated a weakly but significant and independent association of serum soluble TRAIL levels with central obesity, represented by waist circumference, and with LDL-cholesterol. These findings may be the starting point of future longitudinal studies aimed to understand the biological basis of these relationships and to analyze how TRAIL concentrations vary according to body composition changes.
